# Metabonomics Reveals Drastic Changes in Anti-Inflammatory/Pro-Resolving Polyunsaturated Fatty Acids-Derived Lipid Mediators in Leprosy Disease

**DOI:** 10.1371/journal.pntd.0002381

**Published:** 2013-08-15

**Authors:** Julio J. Amaral, Luis Caetano M. Antunes, Cristiana S. de Macedo, Katherine A. Mattos, Jun Han, Jingxi Pan, André L. P. Candéa, Maria das Graças M. O. Henriques, Marcelo Ribeiro-Alves, Christoph H. Borchers, Euzenir N. Sarno, Patrícia T. Bozza, B. Brett Finlay, Maria Cristina V. Pessolani

**Affiliations:** 1 Laboratório de Microbiologia Celular, Instituto Oswaldo Cruz, Fundação Oswaldo Cruz, Rio de Janeiro, Brazil; 2 Laboratório de Biologia, Instituto Nacional de Metrologia, Qualidade e Tecnologia, Rio de Janeiro, Brazil; 3 Michael Smith Laboratories, The University of British Columbia, Vancouver, British Columbia, Canada; 4 Escola Nacional de Saúde Pública Sergio Arouca, Fundação Oswaldo Cruz, Rio de Janeiro, Brazil; 5 Centro de Desenvolvimento Tecnológico em Saúde, Fundação Oswaldo Cruz, Rio de Janeiro, Brazil; 6 University of Victoria – Genome BC Proteomics Centre, University of Victoria, Victoria, British Columbia, Canada; 7 Laboratório de Farmacologia Aplicada, Farmanguinhos, Fundação Oswaldo Cruz, Rio de Janeiro, Brazil; 8 Laboratório de Pesquisa em Farmacogenética, Instituto de Pesquisa Clínica Evandro Chagas, Fundação Oswaldo Cruz, Rio de Janeiro, Brazil; 9 Laboratório de Hanseníase, Instituto Oswaldo Cruz, Fundação Oswaldo Cruz, Rio de Janeiro, Brazil; 10 Laboratório de Imunofarmacologia, Instituto Oswaldo Cruz, Fundação Oswaldo Cruz, Rio de Janeiro, Brazil; Institut Pasteur, France

## Abstract

Despite considerable efforts over the last decades, our understanding of leprosy pathogenesis remains limited. The complex interplay between pathogens and hosts has profound effects on host metabolism. To explore the metabolic perturbations associated with leprosy, we analyzed the serum metabolome of leprosy patients. Samples collected from lepromatous and tuberculoid patients before and immediately after the conclusion of multidrug therapy (MDT) were subjected to high-throughput metabolic profiling. Our results show marked metabolic alterations during leprosy that subside at the conclusion of MDT. Pathways showing the highest modulation were related to polyunsaturated fatty acid (PUFA) metabolism, with emphasis on anti-inflammatory, pro-resolving omega-3 fatty acids. These results were confirmed by eicosanoid measurements through enzyme-linked immunoassays. Corroborating the repertoire of metabolites altered in sera, metabonomic analysis of skin specimens revealed alterations in the levels of lipids derived from lipase activity, including PUFAs, suggesting a high lipid turnover in highly-infected lesions. Our data suggest that omega-6 and omega-3, PUFA-derived, pro-resolving lipid mediators contribute to reduced tissue damage irrespectively of pathogen burden during leprosy disease. Our results demonstrate the utility of a comprehensive metabonomic approach for identifying potential contributors to disease pathology that may facilitate the development of more targeted treatments for leprosy and other inflammatory diseases.

## Author Summary

Leprosy is caused by a mycobacterium that has a predilection for the skin and nerve cells, and the disease is treated with a combination of antibiotics (multidrug therapy, MDT). Nerve damage caused by the infection may lead to permanent disabilities, and can happen even during MDT and subsequent to patient release. Therefore, a more comprehensive understanding of the interaction between the leprosy bacillus and humans is mandatory in order to develop new tools for better disease control and management. Aiming to understand more about the effects of leprosy on human metabolism, we analyzed the chemical composition of sera from leprosy patients before and after MDT. Our results show that specific classes of molecules are affected by the infection, and that MDT can partially reverse these effects. In particular, lipids related to polyunsaturated fatty acid metabolism and known to play a role in the host's defense mechanisms were highly affected during the disease. A complete understanding of all the steps in this process may open new avenues for leprosy treatment with consequent prevention of neuropathy.

## Introduction

Leprosy, a chronic infectious disease caused by the obligate intracellular bacterium *Mycobacterium leprae*, remains a major source of morbidity in developing countries [1]. The disease affects mainly the skin and the peripheral nervous system, in which the leprosy bacillus is preferentially found inside macrophages and Schwann cells (reviewed in [2]). Multidrug therapy (MDT), a combination of antibiotics that are very effective in eliminating *M. leprae*, was introduced by WHO in the early eighties. However, despite efforts to treat registered leprosy patients, the number of new cases reported globally remains stable and high (about 200,000/year). The disease is still considered a public health problem in several countries. In Brazil, the detection rate remains high and stable at approximately 40,000 new cases annually [1]. Moreover, nerve damage may progress during MDT itself and even subsequent to patient release, due mainly to the occurrence of acute immune-inflammatory episodes known as leprosy reactions. Therefore, new strategies and approaches need to be developed in order to decrease disease morbidity and fully eradicate leprosy as a public health problem.

Also known as Hansen's disease, leprosy manifests as a spectrum of clinical forms in correlation with the nature and magnitude of the innate and adaptive immune responses generated during infection. At one extreme of the spectrum, individuals with polar tuberculoid (TT) leprosy have few lesions and manifest a contained, self-limited infection in which scarce bacilli are detected due to the generation of a strong cellular immune response against *M. leprae*. At the other end, lepromatous leprosy (LL) is a progressively disseminating disease characterized by extensive bacterial multiplication within host cells and low cell-mediated immunity to the pathogen. Between these two poles are the borderline forms (characterized by their intermediate clinical and immunological patterns), commonly referred to as borderline tuberculoid (BT), borderline borderline (BB), and borderline lepromatous (BL) in accordance with their proximity to either one of the spectral extremes (reviewed in [2]).

Leprosy is a complex disease, and is essentially restricted to human beings. Despite considerable research efforts over the last decades, our understanding of the mechanisms that govern leprosy pathogenesis remains limited. The unique features of the leprosy bacillus have contributed to the slow progress in our knowledge of leprosy. One peculiar characteristic of *M. leprae* is its extremely long generation time, estimated to be nearly 2 weeks. This slow growth rate results in long incubation periods (2–10 years) and very slow development of pathology and clinical evolution (reviewed in [2]). In the absence of an animal experimental model that mimics the disease in humans, progress in our knowledge of leprosy pathogenesis relies on observations obtained from infected populations and on analyses of clinical samples collected directly from leprosy patients. However, continuing improvements in analytical technologies and recent developments of sensitive high-throughput techniques are now opening a new opportunity to study this ancient disease in order to suggest new strategies for leprosy prevention and treatment. Of note, techniques that identify and quantify multiple small metabolites (<1,500 Da) in complex biological samples have been recently developed, giving rise to the field of metabolomics (or metabonomics). Metabonomics has been successfully applied to different biofluids and tissue types, revealing their biochemical composition in different pathological conditions [3], [4], [5].

The complex interplay between pathogens and their hosts has profound effects on host metabolism during infection. Since the tuberculoid and lepromatous forms of leprosy constitute different responses of the host to *M. leprae* infection, we hypothesized that host metabolism in response to infection would be distinct in these different clinical forms of the disease. Even though *M. leprae* is an obligate intracellular parasite, patient plasma/serum offers an important window for detecting metabolic modulation since blood contains many molecules that are released by different tissues in response to infection. A recent metabolomic study of human serum has identified and quantified more than 4,000 metabolites generating the Human Serum Database [6].

To explore the perturbations in the human metabolome associated with *M. leprae* infection, we analyzed the repertoire of metabolites present in serum samples of leprosy patients. We used direct-infusion ultrahigh resolution Fourier transform ion cyclotron resonance mass spectrometry (DI-FT-ICR-MS), a powerful technique that allows the presumptive identification and relative quantification of thousands of metabolites with high mass accuracy and without the need for extensive sample preparation [7]. Our results indicate a marked modulation of omega-6 and omega-3 polyunsaturated fatty acids (PUFA) metabolism during *M. leprae* infection, which disappears after MDT. Effects of *M. leprae* infection on PUFA metabolism were confirmed by measurements through enzyme-linked immunoassays using serum, which showed significantly higher levels of prostaglandin (PG) D_2_ and E_2_ (PGD_2_ and PGE_2_), lipoxin A_4_ (LXA_4_) and resolving D_2_ (RvD_2_) in untreated leprosy patients. Moreover, high-throughput metabolic profiling of skin specimens revealed an abundance of lipase products in LL patients, such as polyunsaturated fatty acids and lysolecithin, corroborating the serum metabolome data. This study demonstrates the power of metabonomics to unravel metabolic modulation during infection and provides the opportunity to identify novel therapeutic targets and biomarkers for leprosy.

## Materials and Methods

### Ethics statement

The Ethics Committee of the Oswaldo Cruz Foundation approved all procedures described in this study. All subjects, none of which were minors, provided informed written consent.

### Patients and specimens

Leprosy patients (29 LL and 29 BT) were recruited on a volunteer basis from the Leprosy Outpatient Unit (Oswaldo Cruz Foundation, Rio de Janeiro, RJ, Brazil). Patients were classified with leprosy according to the criteria of Ridley and Jopling [8], and serum samples were taken before and right after MDT conclusion (without fasting). Skin biopsy specimens (6-mm punch) were also collected from LL and BT patients before treatment and were used for metabolite extraction. The baseline characteristics of each group of individuals included in the study are shown in Table 1. None of the patients were under anti-inflammatory therapy at the time of serum and biopsy specimen collection.

**Table 1:** Baseline characteristics of leprosy patients and healthy controls.

Clinical sample	Sera					Skin biopsies	
Group	Control	BT		LL		BT	LL
Method^a^	EIA	FTICRMS	EIA	FTICRMS	EIA	FTICRMS	FTICRMS
Individuals (n)	10	4	25^c^	4	25^d^	4	4^e^
Male	6	3	9	3	18	2	2
Female	4	1	16	1	7	2	2
Age (median)	34.2	50.5	48	61.5	50	44	75
Age (min-max)	18–52	32–56	11–66	50–87	22–87	31–63	56–88
BI (median)^b^	-	0	0	3.91	4.33	0	4.25

Groups included in this study: controls; BT, borderline tuberculoid patients; LL, lepromatous patients.

a EIA, enzyme-linked immunoassay; FTICRMS, Fourier transform ion cyclotron resonance mass spectrometry.

b BI, baciloscopic index.

c Four of these patients underwent Type I reaction during treatment.

d Nine patients developed Type II reaction and two developed Type I reaction.

e One patient underwent Type II reaction.

### Metabolite extraction

Serum samples were thawed and 200 µL of serum were extracted overnight at −20°C in 2-mL tubes with 750 µL of methanol/chloroform (2∶1, v/v) followed by vortexing and centrifugation at 1,500×*g* for 5 minutes at 4°C. It is important to note that serum samples were never thawed before the metabonomics analysis described below was performed. The supernatants were carefully transferred to new tubes and the samples were extracted once again with 500 µL of methanol/chloroform/water (2∶1∶0.8, v/v/v), vortexed, and centrifuged as described before [7]. The extracts were pooled, concentrated in a speedvac evaporator and dried under a nitrogen stream. For metabolite extraction of frozen biopsies, specimens were thawed on ice, mechanically disrupted, and extracted with chloroform/methanol/water (1∶2∶0.8, v/v/v) [9]. Samples were then partitioned with chloroform and methanol (2∶1, v/v), according to the standard procedure of Folch *et al.* (1957) [10]. Pellets were extracted again with acetonitrile (10 µL of acetonitrile for each mg of initial tissue) by vortexing for 10 minutes. Samples were clarified by centrifugation at 16,000×*g* for 5 minutes, all phases were combined and extracts were dried and saved for further analysis.

### Direct Infusion Fourier Transform Ion Cyclotron Resonance Mass Spectrometry (DI-FT-ICR-MS)

For metabolic profiling of sera, dried extracts were suspended in 70% methanol (100 µL for each µL of sample), vortexed, and cleared by centrifugation. Supernatants were collected and used as described below. Extracts were diluted 1∶3 with 70% methanol containing either 0.2% formic acid (for positive ionization mode) or 0.2% ammonium hydroxide (for negative ionization mode) and spiked with predefined amounts of an ES tuning mix solution as the internal standard for mass calibration. For metabolic profiling of skin biopsies, dried extracts were resuspended in 60% acetonitrile (100 µL per 10 mg of sample), vortexed, sonicated and cleared by centrifugation. Extracts were diluted 1∶3 in ESI standard solutions containing either 0.2% formic acid (positive ion mode) or 0.5% ammonium hydroxide (negative ion mode). Solutions were then infused, using a syringe pump (KDS Scientific, Holliston, MA), at a flow rate of 2.5 µL per minute, into a 12-T Apex-Qe hybrid quadrupole-FT-ICR mass spectrometer (Bruker Daltonics, Billerica, MA) equipped with an Apollo II electrospray ionization source, a quadrupole mass filter, and a hexapole collision cell. Data were recorded in positive and negative ion modes with broadband detection and an FT acquisition size of 1,024 kilobytes per second within an *m/z* range of 150 to 1,100. Other experimental parameters were: capillary electrospray voltage of 3,600 to 3,750 V, spray shield voltage of 3,300 to 3,450 V, source ion accumulation time of 0.1 second, and collision cell ion accumulation time of 0.2 second. To increase detection sensitivity, survey scan mass spectra in positive- and negative-ion modes were acquired from the accumulation of 200 (sera) or 400 (skin) scans per spectrum.

### DI-FT-ICR-MS data processing

Raw mass spectrometry data were processed using a custom-developed software package, as described elsewhere [7]. Then, data analysis proceeded as previously described [3], [11]. Principal Component Analysis (PCA) was performed using the freely available software Multibase (http://www.numericaldynamics.com/). To identify differences in metabolite composition between BT and LL sera and skin samples, and sera from both groups before and after MDT, we manually selected two groups of metabolites. The first group comprised metabolites that were present in one set of samples but not the other. The second group comprised metabolites present in the two sets of samples being compared, but at different levels. To identify the metabolites in the second group, we averaged the mass intensities of metabolites in each set of samples (BT or LL, before or after MDT) and calculated the ratios between averaged intensities of metabolites from those samples. To assign possible metabolite identities to *m/z* values present in one group of samples but not the others as well as those *m/z* showing at least a 2-fold change in intensity between sets of samples, *m/z* of interest were queried against MassTRIX (version 2, http://metabolomics.helmholtz-muenchen.de/masstrix2/), a free-access software designed to incorporate masses into metabolic pathways using the Kyoto Encyclopedia of Genes and Genomes (KEGG) database (http://www.genome.jp/kegg/).

### Lipoxin A_4_, prostaglandin D_2_, prostaglandin E_2_, leukotriene B_4_ and resolvin D1 measurements

Lipoxin A_4_ (LXA_4_), prostaglandin D_2_ (PGD_2_), prostaglandin E_2_ (PGE_2_), leukotriene B_4_ (LTB_4_) and resolvin D1 (RvD1) levels were measured in serum samples taken from BT (n = 20) and LL (n = 19) patients prior to and after MDT. In addition, measurements of these eicosanoids in serum samples from 10 healthy controls were taken for comparison. We used serum samples that had never been thawed and had been stored at −20°C. Measurements were performed using commercially-available kits according to the manufacturer's instructions. PGD_2_, PGE_2_, LTB_4_ and RvD1 enzyme-linked immunoassay (EIA) kits were purchased from Cayman Chemical (Ann Arbor, USA). The LXA_4_ EIA kit was from Neogen (Lexington, USA). LXA_4_ and RvD1 were extracted from serum samples using C_18_ Sep-Pak columns (Waters; Elstree, UK) before analysis, following the manufacturer's protocol.

### Statistical analysis

Data were analyzed by two-tailed unpaired or paired *t* tests with 95% confidence intervals or Kruskall–Wallis non-parametric analysis of variance (ANOVA) and Dunn's multiple-range post hoc test, as indicated. Outliers were detected using the Grubbs' test and removed from data sets when indicated.

## Results

### BT and LL leprosy patients exhibit distinct serum metabolite profiles

**Figure 1:**
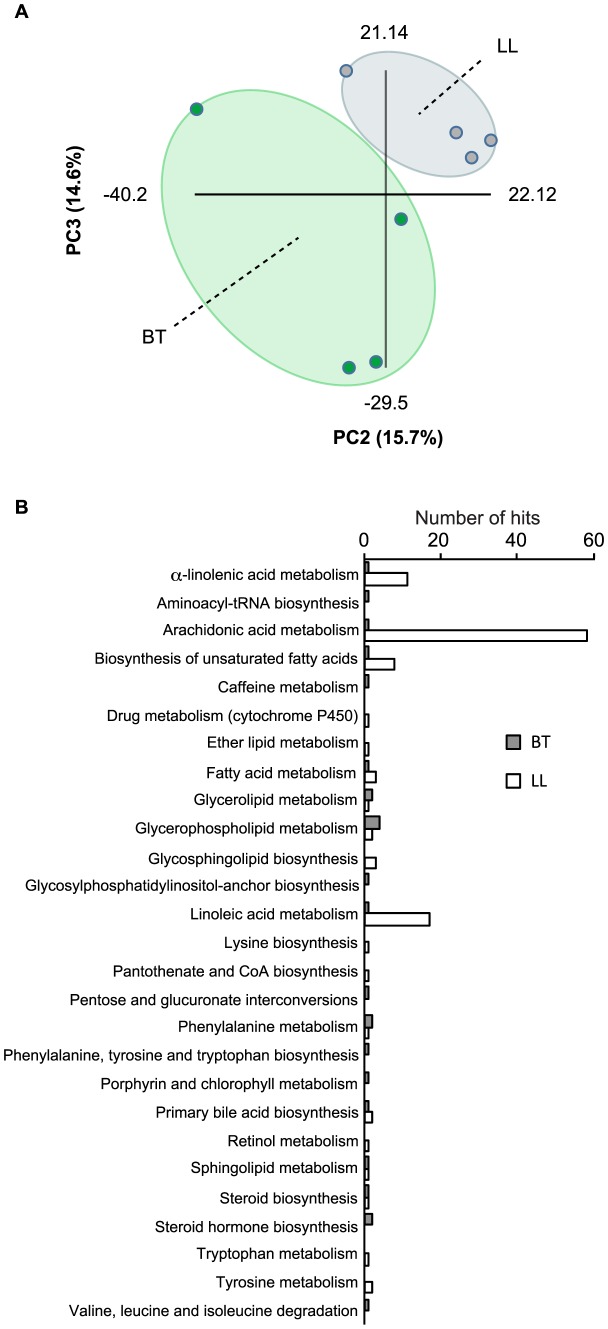
Metabonomics analysis of sera from leprosy patients. (a) Principal component analysis of metabolic alterations on sera from borderline tuberculoid (BT) and polar lepromatous leprosy (LL) patients. Raw DI-FT-ICR-MS data in both negative and positive ionization modes were combined and PCA was performed using Multibase (http://www.numericaldynamics.com/). Sample groups are indicated by the dashed lines. (b) Metabolic pathways altered in the polar forms of leprosy. *m/z* of interest detected in both negative and positive ionization modes were searched against the KEGG database (http://www.genome.jp/kegg/) using the MassTRIX software (version 2, http://metabolomics.helmholtz-muenchen.de/masstrix2/). Bars indicate the number of metabolic features from each KEGG pathway that was affected by infection. Gray bars represent the number of metabolic features that were found in higher levels in BT patients (>2 fold), whereas white bars represent the metabolic features found in higher levels in LL patients.

Serum samples were obtained from 4 BT and 4 LL patients to analyze metabolic alterations during the course of leprosy, and DI-FT-ICR-MS was used to detect and relatively quantify small metabolites in these samples. Such high-throughput analysis yielded a total of 2,565 different *m/z* (metabolite features) from both BT and LL groups, which were detected from combined positive and negative ion modes (Table S1). A Principal Component Analysis plot (Figure 1A) illustrates the extensive differences in metabolic composition between sera from BT and LL patients. Due to the sample size no analysis could be done on gender and age. Nevertheless, a more refined analysis was then carried out to determine the extent of the metabolic differences between BT and LL leprosy patients. To investigate which of the metabolites detected in both BT and LL samples were present at different levels in these groups, the average intensities of all metabolites were calculated and results from each of the sample groups (BT and LL) were compared. Metabolites that showed changes of 2-fold or more were used for further analyses. Based on this analysis criterion, we found that 684 of the total 2,565 metabolites were present at different levels when comparing samples from BT and LL patients (Table S1). This represents 26.7% of all detected *m/z*, supporting the notion that an extensive metabolic shift occurs during disease. Metabolite levels were affected to various degrees, with changes ranging from 2-fold to over 30-fold. The complete serum DI-FT-ICR-MS raw data set is shown in Tables S2 and S3.

### Polyunsaturated fatty acid metabolites are increased in sera from LL patients when compared to BT patients

In order to identify the metabolic pathways most significantly disturbed during leprosy, we selected metabolites detected in both BT and LL patients showing at least a 2-fold difference between them and queried the MassTRIX database (version 2, http://metabolomics.helmholtz-muenchen.de/masstrix2/) to determine putative metabolite identities. Figure 1B shows the categories of metabolites that differ between the two groups. Although many metabolic pathways were affected, our data suggest that the metabolism of omega-6 (linoleic and arachidonic acids) and omega-3 PUFAs (α-linolenic acid, EPA and DHA) are markedly modulated during *M. leprae* infection, with higher levels of a diverse class of bioactive lipid mediators in LL sera when compared to BT sera. The effects of *M. leprae* infection on specific classes of PUFAs are described in more detail below.

#### Arachidonic acid metabolism

The arachidonic acid (AA) pathway was the metabolic route with the highest number of metabolites having increased levels in sera of LL patients (58 potential metabolites) when compared to BT patients. On the other hand, only one putative metabolite in this pathway showed increased levels in BT patients when compared to LL patients. Figure S1 shows the human metabolic pathway of AA as it appears in KEGG, indicating the potential metabolites affected in LL serum. Besides AA itself (*m/z* 303.23292), several *m/z* corresponding to an array of potential AA derivatives were detected in higher levels (two fold or higher) in LL sera. These *m/z*, together with their corresponding potential identities and relative levels detected in LL versus BT patients are shown in Table 2. Although statistical analysis did not yield significant *p*-values (*p*>0.05), this is not surprising due to the limited number of samples used and the inherent variability amongst human samples. Nevertheless, the high number of metabolites in this pathway that were identified by our exploratory method suggests that this is indeed an important component of the host's response to *M. leprae* infection.

**Table 2:** Comparison of the relative levels^a^ of metabolites of the arachidonic acid pathway in sera from BT and LL patients before and after antibiotic treatment.

*m/z*	Compound	BT		LL	
[M-H]^−^		Before	After	Before	After
303.23292	arachidonic acid	100%(38)	56.0%(18)	261.6%(190)	55.8%(12)
317.21222	5-oxo-ETE	100%(45)	33.4%(14)	292.8%(199)	25.8%(7)
	12-oxo-ETE				
	15-oxo-ETE				
	LTA_4_				
319.22782	EETs	100%(41)	34.0%(24)	654.4%(537)	23.0%(8)
	HETEs				
	5-HETE				
	15(R)-HETE				
	15(S)-HETE				
	19(S)-HETE				
	20-HETE				
325.20202	2,3-dinor-8-iso-PGF_2α_	100%(19)	102.7%(44)	352.1%(246)	120.0%(55)
327.21782	2,3-dinor-8-iso-PGF_1α_	100%(21)	189.6%(100)	513.7%(314)	246.1%(134)
333.20712	PGA_2_	100%(58)	112.4%(16)	699.0%(511)	80.8%(38)
	PGB_2_				
	PGC_2_				
	PGJ_2_				
	Δ^12^-PGJ_2_				
	12-keto-LTB_4_				
	5,6-epoxy-tetraene				
335.22282	HPETEs	100%(48)	26.9%(17)	367.6%(275)	21.0%(9)
	5-HPETE				
	15(S)-HPETE				
	hepoxilin A_3_				
	hepoxilin B_3_				
	11H-14,15-EETA				
	15H-11,12-EETA				
	LTB_4_				
	20-OH-LTB_4_				
337.23842	DHETs	100%(27)	80.9%(30)	443.0%(342)	34.1%(10)
351.21782	TXA_2_	100%(60)	25.1%(11)	245.9%(163)	22.4%(8)
	prostacyclin				
	PGD_2_				
	PGE_2_				
	PGH_2_				
	15-keto-PGF_2α_				
	LXA_4_				
	LXB_4_				
369.22832	TXB_2_	100%(60)	33.7%(9)	405.1%(273)	44.7%(16)
	6-keto-PGF_1α_				

a Absent values were substituted with the limit of detection, represented by the lowest intensity value of any given sample. Then, averaged values from the untreated BT serum samples (n = 4) were normalized to 100% and other samples were normalized accordingly. SD are shown in parentheses. PG, prostaglandin; LT, leukotriene; TX, thromboxane; EET, epoxyeicosatrienoic acid; oxo-ETE, oxoicosatetraenoic acid; HETE, hydroxyeicosatetraenoic acid; HPETE, hydroperoxyeicosatetraenoic acid; DHET, dihydroxyeicosatrienoic acid.

Through the action of cyclooxygenases (COX), AA is transformed to PGG_2_ and then to PGH_2_, which may be metabolized to other PGs (PGD_2_, PGE_2_, PGI_2_ and PGF_2α_) and thromboxanes (TXA_2_ and TXB_2_). Several of these compounds, as well as their downstream products, were found in higher levels in LL sera, suggesting high activity of cyclooxygenases during *M. leprae* infection. PGF_2α_ is an important AA metabolite involved in chronic and acute inflammation. It has a local action and is quickly degraded to 15-keto-PGF_2α_ upon reaching the bloodstream. Levels of this metabolite are used as a parameter to measure the *in vivo* biosynthesis of PGF_2α_, as well as its release (reviewed in [12]). An increase in the relative levels of a compound of *m/z* [M-H]^−^ 351.21782, which may correspond to 15-keto-PGF_2α_ (among others), was observed in LL patients (Table 2). TXA_2_ and PGI_2_ are substances with opposite effects: while TXA_2_ is produced by platelets, has vasoconstrictive properties, and stimulates platelet aggregation, PGI_2_ is a vasodilator synthesized by macrovascular endothelial cells that inhibits platelet activation, modulating platelet-vascular interactions [13]. TXA_2_ and PGI_2_ have the same molecular mass (*m/z* [M-H]^−^ 351.21782), and the same is true for their respective degradation products, TXB_2_/6-keto-PGF_1α_ (*m/z* [M-H]^−^ 369.22832) and 11-dehydro-TXB_2_/6-keto-PGE_1_ (*m/z* [M-H]^−^ 367.21282). All of these *m/z* were present in higher levels in LL sera (Table 2).

Another fate of AA is its metabolism by lipoxygenases (5-, 12- or 15-LOX), leading to the formation of LTs, LXs and hepoxilins (HX). Hydroperoxyeicosatetraenoic acids (5-, 12-, or 15-HPETEs), hydroxyeicosatetraenoic acids (5-, 12- or 15-HETEs) and oxo-ETEs are bioactive products of these pathways (reviewed in [14]). Several of these compounds and their degradation products such as DHETs were found in higher levels in LL sera, suggesting a high activity of LOXs during *M. leprae* infection (Table 2). Peroxidation of AA occurs in the presence of free radicals, leading to the non-enzymatic formation of 8-isoprostane (8-iso-PGF_2α_), which possesses vasoconstrictive activities and induces the COX-mediated formation of PGF_2α_. 8-isoprostane has a short half-life, being metabolized to 2,3-dinor-8-iso-PGF_2α_ and 2,3-dinor-5,6-dihydro-8-iso-PGF_2α_, among others (reviewed in [12]). In sera from LL patients, higher levels of 2,3-dinor-8-iso-PGF_2α_, but not of 8-isoprostane, were observed (Table 2), which could be linked to the higher oxidative stress and lower antioxidant capacity observed in leprosy, more specifically in patients with the lepromatous form [15], [16].

#### Linoleic acid metabolism

Linoleic acid may be converted to AA or suffer epoxygenation, generating an array of different biologically active products [17]. As shown in Table 3 and Figure S2, an *m/z* that may correspond to linoleic acid (*m/z* 279.23282) as well as other linoleic acid derivatives was found in higher levels in LL sera (Table 3). These include the 9,10- and 12,13-epoxyoctadecamonoenoic acids (9(10)-EpOME and 12(13)-EpOME, respectively; *m/z* 295.22782), also known as leukotoxins, and which are generated from activated neutrophils and macrophages and can induce cell death. The toxic effects of these metabolites are thought to be mediated by their conversion into the corresponding diols 9,10- and 12,13-diHOME by soluble epoxide hydrolase [18]. On the other hand, one *m/z* that may correspond to a lecithin was found in higher levels in BT sera (*m/z* 704.52438, data not shown).

**Table 3:** Comparison of the relative levels^a^ of metabolites of the linoleic acid pathway in sera from BT and LL patients before and after antibiotic treatment.

*m/z*	Compound	BT		LL	
[M-H]^−^		Before	After	Before	After
277.21732	γ-linolenate	100%(27)	63.8%(23)	200.5%(107)	135.2%(29)
	crepenynate				
279.23282	linoleate	100%(33)	61.2%(23)	310.8%(227)	110.9%(22)
	9-*cis*,11-*trans*-octadecadienoate				
293.21222	9-oxoODE	100%(35)	71.4%(33)	1,720.1%(1,582)	126.9%(63)
	13-oxoODE				
295.22782	9(S)-HODE	100%(56)	3.7%(0)	1,962.6%(1,841)	77.7%(45)
	12(13)-EpOME				
	9(10)-EpOME				
	13(S)-HODE				
329.23342	9,12,13-TriHOME	100%(19)	175.8%(112)	1,197.8%(997)	291.9%(229)
	9,10,13-TriHOME				
	9,10-dihydroxy-12,13-epoxy-octadecanoate				
826.66842	lecithin	100%(22)	188.1%(37)	215.0%(48)	267.5%(22)

a Absent values were substituted with the limit of detection, represented by the lowest intensity value of any given sample. Then, averaged values from the untreated BT serum samples (n = 4) were normalized to 100% and other samples were normalized accordingly. SD are shown in parentheses. EpOME, epoxyoctadecenoic acid; HODE, hydroxyoctadecadienoic acid; TriHOME, trihydroxyoctadecenoic acid; oxoODE, oxooctadecadienoic acid.

Linoleic acid can also be hydroxylated by both CYP and LOX to form HODEs, which can subsequently be oxidized to form oxoocta-decadienoic acids (oxoODEs). Compounds with *m/z* 293.21222 and *m/z* 295.22782, corresponding to potential oxoODEs and HODE, respectively, were detected in higher levels in sera from LL patients (Table 3). These metabolites have been shown to be potent activators of peroxisome proliferator-activated receptor gamma, PPAR-γ [19]. As with metabolites in the arachidonic acid pathway, statistical analysis of the results obtained with metabolites of linoleic acid metabolism did not yield significant *p*-values (*p*>0.05) due to the limited number of samples used and the inherent variability amongst human samples. Nevertheless, the overrepresentation of this pathway in our metabonomics analysis suggests that it is also an important player in the pathophysiology of leprosy.

#### Omega-3 polyunsaturated fatty acid metabolism

Finally, higher relative amounts of potential metabolites derived from the omega-3 polyunsaturated fatty acids were observed in sera from LL patients when compared to sera from BT patients, as seen in Table 4. Again, statistical analysis did not yield significant *p*-values (*p*>0.05) due to the reasons mentioned previously but many metabolites of this pathway showed relative changes in the different patient groups, suggesting that this may be another important player during leprosy that should be investigated further. The α-linolenic acid metabolism in humans and the potential metabolites derived from omega-3 fatty acids with increased levels in LL in comparison to BT are indicated in Figure S3. Recently, the metabolism of omega-3 fatty acids, more specifically eicosapentaenoic acid (EPA) and docosahexaenoic acid (DHA), has received considerable attention due to their anti-inflammatory, pro-resolving activities. EPA and DHA can be obtained directly from the diet or from enzymatic conversions of linolenic acid. Metabolites with masses corresponding to EPA (*m/z* 301.21732) and DHA (*m/z* 327.23302) were found in BT and LL sera, with higher levels being found in sera of LL patients. Both EPA and DHA are metabolized to 3-series PGs and TXs, 5-series LTs, E- and D-series resolvins (RvE and RvD), aspirin D-series resolvins, (neuro)protectin D1 (PD1) and maresins (MaR), respectively, which exert anti-inflammatory and pro-resolution effects (reviewed in [20]). Interestingly, particularly higher levels of *m/z* 333.20712 and 375.21792, which may correspond to RvE2 and RvD1-RvD4, respectively, were detected in sera from LL patients (Table 4). Of note, higher levels of *m/z* 359.22292, which may correspond to 6 different metabolites [RvD5, RvD6, (N)PD1, MaR1, 17-HpDHA (a marker of the D-series Rvs and (N)PD1 biosynthetic pathway) and 14-HpDHA (an intermediate in the synthesis of MaR1)] were also found in sera from LL patients. These data suggest that the metabolism of PUFAs is severely disturbed during leprosy; our data suggest that the production of anti-inflammatory, pro-resolving Rvs, PD1 and MaR derived from omega-3 fatty acid metabolism is highly exacerbated during leprosy, especially in its lepromatous form.

**Table 4:** Comparison of the relative levels^a^ of omega-3 polyunsaturated fatty acids derivates in sera from BT and LL patients before and after antibiotic treatment.

*m/z*	Compound	BT		LL	
[M-H]^−^		Before	After	Before	After
277.21732	α-linolenic acid	100%(27)	63.8%(23)	200.5%(107)	135.2%(29)
301.21732	EPA	100%(30)	51.8%(20)	253.4%(153)	38.4%(24)
305.24862	ETA	100%(37)	60.2%(18)	198.0%(145)	54.1%(12)
317.21222	18-HEPE	100%(46)	33.4%(14)	292.8%(200)	25.8%(7)
327.23302	DHA	100%(36)	74.2%(35)	199.0%(100)	95.5%(35)
333.20712	RvE2	100%(58)	112.4%(16)	699.0%(512)	80.8%(38)
349.20212	RvE1	100%(49)	37.1%(9)	193.0%(98)	30.8%(3)
359.22292	17-HpDHA	100%(41)	31.8%(11)	686.3%(539)	34.7%(11)
	14-HpDHA				
	RvD5,RvD6				
	(N)PD1				
	MaR1				
375.21792	RvD1-RvD4	100%(48)	41.3%(16)	400.0%(265)	28.4%(4)

a Absent values were substituted with the limit of detection, represented by the lowest intensity value of any given sample. Then, averaged values from the untreated BT serum samples (n = 4) were normalized to 100% and other samples were normalized accordingly. SD are shown in parentheses. EPA, eicosapentaenoic acid; ETA, eicosatetraenoic acid; DHA, docosahexaenoic acid; RvE, E-series resolvins; HpDHA, hydroperoxydocosahexaenoic acid; RvD, D-series resolvins; (N)PD1, (neuro)protectin D1; MaR1, maresin 1. HEPE, hydroxyeicosapentaenoic acid.

### Multidrug therapy converts both BT and LL groups to a common polyunsaturated fatty acid metabolic phenotype

**Figure 2:**
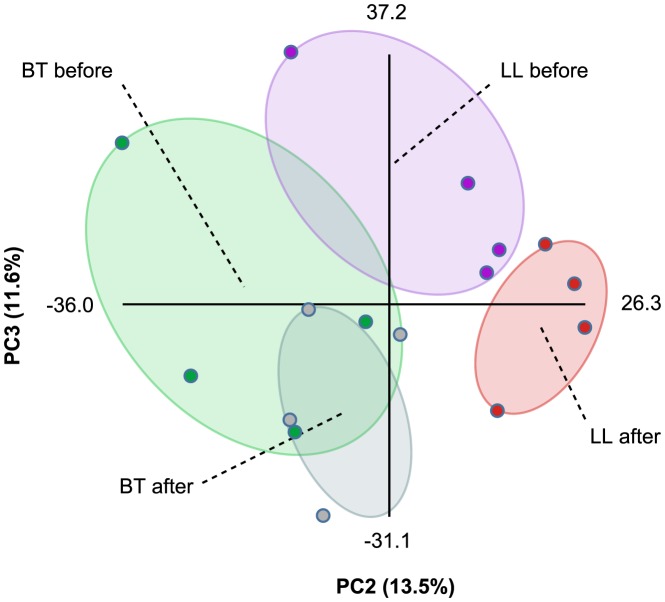
Principal component analysis of the metabonomics data. Raw DI-FT-ICR-MS data in both negative and positive ionization modes were combined and PCA was performed using Multibase (http://www.numericaldynamics.com/). Plots show the separation of groups based on the pole of disease (BT, LL) and treatment status (before, after). Sample groups are indicated by the dashed lines.

In order to gain further insights into the metabolic changes elicited during leprosy, sera samples from the same patients were collected before and immediately after the conclusion of MDT (six and twelve months for BT and LL patients, respectively). Total metabolites were extracted and analyzed by DI-FT-ICR-MS as described above. As an attempt to compare the metabolic profiles of the four groups of samples, we performed Principal Component Analysis (PCA) on this dataset using Multibase (http://www.numericaldynamics.com/). As can be seen from Figure 2, such analysis showed a clear separation between the BT and LL groups. Also, the PCA showed a clear separation of the LL samples before and after MDT, although the separation of BT samples before and after treatment was modest. This is in line with more extensive effects of lepromatous leprosy on host metabolism due to the high bacillary burden. Nevertheless, due to the extensive effects of lepromatous leprosy on polyunsaturated fatty acid metabolism, we focused our analysis on the effect of MDT on this metabolic pathway in both BT and LL patients. MDT caused a decrease in the levels of most potential metabolites from the arachidonic acid pathway, both in the BT and LL groups (Table 2 and Figure S4). This suggests that, although higher levels of these metabolites were generally observed in LL samples when compared to BT, these molecules were present at increased levels in leprosy patients in general, both LL and BT. In contrast, potential metabolites derived from linoleic and α-linolenic acids were mostly affected by MDT only in LL patients, returning to levels similar to those originally found in BT patients (Tables 3 and 4). The more extensive effect of MDT on the metabolic profiles of LL patients supports our initial findings that samples from LL patients show higher levels of eicosanoids and other polyunsaturated fatty acid metabolites than samples from BT patients, and this correlates well with the bacillary burden observed in these clinical forms of leprosy. As shown in Tables 2–4, with the exception of a few *m/z*, relative levels of most metabolites were indistinguishable when comparing BT and LL samples after MDT. In other words, MDT converted BT and LL patients to a common phenotype regarding the metabolic profiles of PUFAs.

### Circulating levels of eicosanoids are altered in leprosy patients

**Figure 3:**
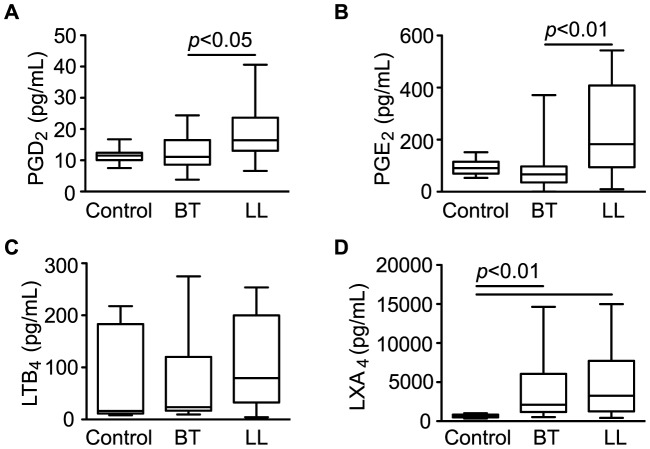
Serum levels of eicosanoids in borderline tuberculoid and polar lepromatous patients determined by EIAs. Box-plots represent serum levels of PGD_2_ (a), PGE_2_ (b), LTB_4_ (c) and LXA_4_ (d) assessed in healthy controls, BT and LL patients, as indicated. Median values are indicated by lines. Outliers were detected using the Grubbs' test and removed. Group comparisons were evaluated with Kruskall–Wallis non-parametric analysis of variance (ANOVA) and Dunn's multiple-range post hoc test. PGD_2_, prostaglandin D_2_; PGE_2_, prostaglandin E_2_; LTB_4_, leukotriene B_4_; LXA_4_, lipoxin A_4_. *P*-values higher than 0.05 are not shown.

**Figure 4:**
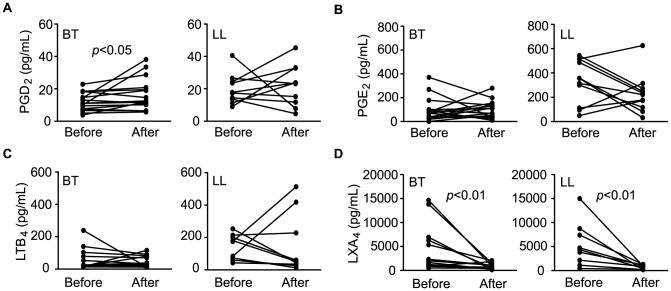
Circulating levels of eicosanoids in borderline tuberculoid and polar lepromatous patients before and after MDT determined by EIAs. Paired values of serum concentrations of PGD_2_ (a), PGE_2_ (b), LTB_4_ (c) and LXA_4_ (d) from each patient, as assessed in BT and LL patients before and right after MDT conclusion. Each line represents one patient. Paired t tests were used for statistical analysis. PGD_2_, prostaglandin D_2_; PGE_2_, prostaglandin E_2_; LTB_4_, leukotriene B_4_; LXA_4_, lipoxin A_4_. *P*-values higher than 0.05 are not shown.

Eicosanoids are lipid mediators that play a critical role as regulators of inflammation and the immune response generated during infection, including those caused by mycobacteria [21], [22], [23], [24], [25], [26]. Among the potential eicosanoids altered during leprosy, several of them possess the same molecular mass. In order to confirm the modulation of some of these compounds during *M. leprae* infection, levels of PGE_2_, PGD_2_, LXA_4_ (*m/z* [M-H]^−^ 351.21782) as well as LTB_4_ (*m/z* [M-H]^−^ 335.22282) were screened by EIAs. Circulating levels of these mediators were determined in leprosy patients (BT, n = 25; LL, n = 25) and compared with their levels in healthy controls (n = 10). While no differences in LTB_4_ levels were detected between different sample groups, the levels of PGD_2_ and PGE_2_ were significantly higher in LL patients when compared to BT (Figure 3), thus confirming the original observation that *m/z* 351.21782 was found in higher levels in LL serum by DI-FT-ICR-MS analysis (Table 2). Next, to reinforce the notion that the altered production of eicosanoids observed in leprosy patients results from an active modulation by the *M. leprae* infection, serum concentrations of PGE_2_, PGD_2_ and LTB_4_ in sera from BT and LL patients were measured at the conclusion of MDT and compared with the levels observed before treatment. By comparing pre- and post-MDT serum samples taken from the same patients, we observed significantly higher PGD_2_ levels in the BT group after the conclusion of MDT, in contrast to the heterogeneous behavior of this mediator observed in LL patients (Figure 4A). After treatment, the PGD_2_ levels were similar between LL and BT (Figure S5). Regarding levels of PGE_2_, a decrease was observed in most LL patients, although 4 of them showed higher levels after treatment (Figure 4B). In contrast, no changes in PGE_2_ levels were observed in most BT patients after the conclusion of MDT, although 3 patients showed a decrease in its levels (Figure 4B). Even after treatment, PGE_2_ levels were significantly higher in LL versus BT patients (Figure S5). LTB_4_ levels tended to decrease both in LL and BT patients, although 2 LL patients showed higher levels after conclusion of MDT (Figure 4C). Finally, as seen previously in the context of untreated patients, no differences between LTB_4_ serum levels in LL versus BT patients were detected after conclusion of MDT (Figure S5).

We also measured the levels of LXA_4_ in serum samples and found that concentrations of this lipid mediator were significantly altered in leprosy patients when compared to healthy controls. LXA_4_ is likely the major contributor to *m/z* [M-H]^−^ 351.21782, followed by PGE_2_. While PGD_2_, PGE_2_ and LTB_4_ serum levels were below 0.6 ng/mL in most samples from leprosy patients, particularly in untreated LL patients, LXA_4_ levels were much higher, ranging from 2 to 17 ng/mL. As shown in Figure 3D, significantly higher levels of LXA_4_ were detected in both BT and LL patients when compared to the controls, but no significant difference was found between these two groups. However, after treatment, serum LXA_4_ levels in BT and LL patients returned to normal (Figure S5). The decrease in LXA_4_ levels in LL and BT sera after the conclusion of MDT can be clearly seen in paired pre- and post-MDT serum samples taken from the same patients (Figure 4D). LXA_4_ concentrations showed a statistically significant decrease after MDT, with a consistent behavior in all analyzed sera. These data suggest that LXA_4_ is a major contributor of *m/z* [M-H]^−^ 351.21782 and point to a more predominant role of LXA_4_ during leprosy.

### Circulating levels of resolvin D1 are altered in leprosy patients

**Figure 5:**
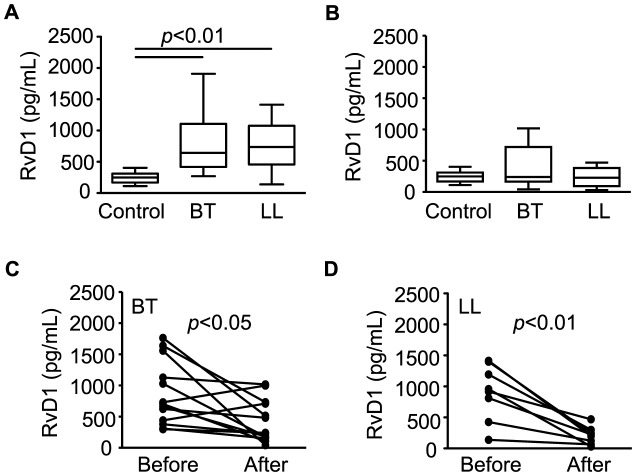
Serum levels of resolvin D1 in borderline tuberculoid and polar lepromatous patients determined by EIAs. Box plots represent serum levels of RvD1 assessed in healthy controls, BT and LL patients before (A) and right after MDT conclusion (B), as indicated. Median values are indicated by lines. Group comparisons were evaluated with Kruskall–Wallis non-parametric analysis of variance (ANOVA) and Dunn's multiple-range post hoc test. Paired values of serum concentrations of RvD1 from each patient before and right after MDT conclusion, as assessed in BT and LL patients, are shown in C and D, respectively. Each line represents one patient. Paired t tests were used for statistical analysis. *P*-values higher than 0.05 are not shown.

Resolvins, including D and E series resolvins, are endogenous lipid mediators generated during the resolution phase of acute inflammation from the omega-3 polyun- saturated fatty acids docosahexaenoic acid (DHA) and eicosapentaenoic acid (EPA), having potent anti-inflammatory and pro-resolution actions in several animal models of inflammation. In order to confirm that the omega-3 polyunsaturated fatty acid metabolism is disturbed during leprosy, levels of RvD1 in sera from leprosy patients were measured by EIA. Circulating levels of this mediator were determined in leprosy patients (BT, n = 20; LL, n = 19) and compared with their levels in healthy controls (n = 6). Interestingly, the results were similar to those observed for LXA_4_, which also has anti-inflammatory and pro-resolution action. Levels of RvD1 were found to be significantly different in leprosy patients when compared to healthy controls (Figure 5A), returning to normal levels after treatment in both BT and LL patients (Figure 5B). The decrease in RvD1 levels in LL and BT sera after the conclusion of MDT can also be seen in paired pre- and post-MDT serum samples taken from the same patients (Figure 5C and D). However, there was no difference between levels of RvD1 between BT and LL sera before MDT (Figure 5A), in contrast to the profile observed for the *m/z* 375.21792 in the DI-FT-ICR analysis (Table 4). Thus, the difference observed in the metabonomic study could be due to other compounds with the same *m/z* such as RvD2–4 or others.

### DI-FT-ICR-MS analysis of skin specimens reveals higher levels of polyunsaturated fatty acid metabolites in LL patients

To expand the metabolite profiles generated with serum samples, we performed a metabolomic analysis of human skin biopsies from 4 cases of LL and 4 cases of BT, looking for alterations in PUFA metabolism at the site of *M. leprae* infection. To do so, we extracted metabolites from the biopsies and analyzed them through DI-FT-ICR-MS, as described above. The complete skin DI-FT-ICR-MS raw data set is shown in Tables S4 and S5. Almost 2,000 metabolites were detected, and their relative abundance was compared between LL and BT lesions. Among the list of *m/z* detected, we selected metabolites showing at least a 2-fold difference between samples from BT and LL patients. As shown in Table 5, *m/z* potentially corresponding to docosapentaenoic acid (DPA), DHA, AA, linoleic acid/9-cis,11-trans-octadecadienoate, 1-acyl-*sn*-glicero-3-phosphocholine (lysolecithin), lecithin and plasmenic acid were present in higher levels in LL lesions. In contrast, the mass 376.2226 Da, which corresponds to several potential metabolites of the arachidonic acid pathway, was present in higher levels in BT lesions. Although definitive metabolite identity cannot be determined using this method, our results suggest that phospholipids and products of PLA_2_ activity accumulate in LL lesions, correlating with the higher levels of potential phospholipids and free unsaturated fatty acids and their derivatives observed in the serum of these patients. Of note, potential DPA (330.2559 Da) levels were about 50 times higher in LL lesions when compared to BT lesions (Table 5). DPA is a 22-carbon PUFA with anti-inflammatory properties derived from an elongation step of EPA, abundantly present in macrophages treated with EPA [27].

**Table 5:** Comparison of the relative levels^a^ of metabolites in skin biopsies from LL and BT patients.

Mass (Da)	Compounds	LL∶BT
330.2559	DPA	50.6∶1
328.2402	DHA	3.4∶1
304.2402	arachidonic acid	2.9∶1
332.2715	adrenic acid	1.9∶1
280.2402	linoleic acid	32.7∶1
	9-*cis*,11-*trans*-octadecadienoate	
703.5152	lecithin	2.8∶1
656.4781	plasmenic acid	6.4∶1
479.3376	1-acyl-*sn*-glycero-3-phosphocoline	8.1∶1
376.2226	8-isoprostane	1∶3.5
	11,14,15-THETA	
	11,12,15-THETA	
	11-epi-PGF_2α_	
	PGF_2α_	
	trioxilin A_3_	
	trioxilin B_3_	

a Data are shown as the ratio of the averaged values from the LL skin samples (n = 4) and the averaged values from the BT skin samples (n = 4). DPA, docosapentaenoic acid; DHA, docosahexaenoic acid; THETA, trihydroxyicosatrienoic acid; PG, prostaglandin.

## Discussion

Although leprosy is one of mankind's oldest diseases, the interplay between the human body and *M. leprae* remains poorly understood. Research in leprosy lacks laboratory tools that can be used to predict susceptibility to the disease and disease progression, which are critical for an improved management of patients through the use of more rational therapeutic approaches. Among the branches of “omics”, the recent development of high-throughput techniques that allow the simultaneous identification and quantification of small metabolites from different tissues and biofluids is emerging as a powerful approach to investigate the modulation of host metabolism during infection, with the perspective to disclose potential contributors to disease pathology. Herein, we have applied a metabonomics analysis of serum samples from leprosy patients to the comparison of host metabolism regulation during infection in two distinct clinical forms of the disease (lepromatous versus tuberculoid). Extensive differences in metabolic composition during leprosy were observed, supporting the notion that a unique metabolic shift occurs during disease. Moreover, serum composition of infected patients converged to a similar profile after conclusion of treatment, indicating that the differences observed resulted from *M. leprae* infection. When analyzing the metabolic pathways affected by *M. leprae*, a robust increase in the levels of potential AA metabolites was observed in LL patients in comparison to BT patients. However, MDT caused a decrease in the levels of most potential metabolites from the arachidonic acid pathway, both in the BT and LL groups. This suggests that, although higher levels of these metabolites were generally observed in LL samples when compared to BT, these molecules were present at increased levels in leprosy patients in general, both LL and BT. One caveat of our metabonomics study is the fact that only a limited number of samples was available for analysis. Therefore, an extensive statistical analysis was not feasible and the results of the metabonomics experiments must be taken with caution. Nevertheless, this approach is very useful in an exploratory mode and many aspects that we have previously investigated using this methodology were confirmed using other techniques [3], [11]. In order to ameliorate this issue, we used EIAs to measure the concentrations of a few molecules of interest in the serum of other leprosy patients and healthy controls. Higher levels of PGD_2_ and PGE_2_ in LL sera when compared to BT sera were confirmed through EIAs. We also found high levels of LXA_4_ in both LL and BT patients in comparison with healthy individuals. Of note was the enrichment of masses that may correspond to omega-3 PUFAs and their biologically-active, anti-inflammatory and pro-resolving Rvs, PD1 and MaR derivatives, some of which were also detected in LL skin lesions. Higher levels of RvD1 were detected by EIA in leprosy patients (both LL and BT), and decreased to normal levels after treatment. To our knowledge, this is the first study reporting the levels of LX and RvD1 during leprosy. The main conclusion of this study is that PUFA metabolism is markedly regulated during *M. leprae* infection, potentially contributing to multiple aspects of the immunopathogenesis of leprosy.

The finding of higher levels of potential free PUFAs both in sera and skin lesions of LL patients, and of lysophosphatidylcholine in LL lesions suggests a high lipid turnover in these lesions. These data agree with previous studies showing a higher expression of host PLA_2_ and PLC in LL patients [28] and of the high PL activity detected in *M. leprae* preparations [29]. PGE_2_ levels were significantly higher in untreated LL patients, returning to levels similar to BT patients after the conclusion of MDT. Accordingly, increased cyclooxygenase-2 expression has been observed in biopsies from LL patients [26], [30]. PGE_2_ is the main cyclooxygenase product produced by macrophages, and it supports acute local inflammation, being at a first moment pro-inflammatory and at the same time immunosuppressive, because it inhibits cell-mediated immunity by selectively inhibiting Th_1_ cytokines (IFN-γ and IL-2) and suppressing IL-12 production in monocytes and dendritic cells (as well as the expression of its receptor), without interfering with the production of the Th_2_ cytokines IL-4 and IL-5. Overproduction of PGE_2_ is observed in Th_2_-associated diseases (asthma, atopic dermatitis) (reviewed in [31]), which is the case of LL leprosy, where humoral immune responses are unable to control the infection. The observed increase in PGE_2_ levels in sera from LL patients agrees with previous studies of PGE_2_ in *M. leprae*, where it was observed in animal models (nude mice) that infected macrophages obtained from footpad granulomas produced high levels of PGE_2_, which was associated with a down-regulation of macrophage and T-cell functions [21]. These functions were restored when PGE_2_ biosynthesis was inhibited, either *in vivo*, when infected mice were subjected to a diet deficient in essential fatty acids, or *in vitro*, by treatment of cultured cells with indomethacin [21], [22]. Human monocytes obtained from LL patients showed a high production of PGE_2_ [24], [32], and other studies showed that the lipid droplets induced in macrophages and Schwann cells by *M. leprae* are sites for PGE_2_ biosynthesis. Moreover, COX-2 was detected in lipid droplets present in nerve and dermal lesions of LL patients, suggesting that they constitute sites of PGE_2_ production *in vivo* [25], [26].

Recent studies indicate that PGE_2_ may have different effects during the course of inflammation. At early stages, as previously described, PGE_2_ presents a pro-inflammatory activity (reviewed in [31]). However, with the progress of the inflammatory process, it was observed that PGE_2_ decreases the production of 4-series LTs through the inhibition of 5-lipoxygenases, and regulates the transcription of 15-lipoxygenase in neutrophils, switching the production of LTs to LXs (reviewed in [20]). Indeed, it has been recently shown that PGE_2_ serves as a feedback inhibitor essential for limiting chronic inflammation in autoimmune arthritis [33]. Furthermore, PGE_2_ inhibits the synthesis of the pro-inflammatory cytokines TNF-α and IL-1 by macrophages (reviewed in [31]). PGE_2_ may undergo a non-enzymatic dehydration reaction, forming the cyclopentenone PGA_2_ and its isomerization products PGC_2_ and PGB_2_. Cyclopentenone PGs have reported anti-inflammatory activity, through activation of PPAR, specifically PPAR-α and PPAR-δ in the case of PGA_2_ (reviewed in [34]). Interestingly, *m/z* 333.20712, which may correspond to PGA_2_, PGB_2_ and PGC_2_, was detected in LL but not BT sera, probably as a consequence of the higher availability of PGE_2_ in LL. Therefore, PGE_2_, in conjunction with its cyclopentenone PG derivatives, may play an immunosuppressive and anti-inflammatory role in LL.

Regarding PGD_2_, it is also a pro-inflammatory eicosanoid, and it elicits inflammatory and vascular responses through interaction with the D prostanoid receptor 1 (DP) and chemoattractant receptor-like molecule expressed on Th_2_ cells (CRTH2). PGD_2_ is capable of inducing chemotaxis of eosinophils, basophils, and Th_2_ cells, stimulating the production of IL-4, IL-5, and IL-13 in the latter [35], and thus eliciting a Th_2_ response, typical of LL immunopathology. Other studies of PGD_2_ synthase (PGDS) expression showed a drop in its biosynthesis after the beginning of the inflammatory process, reaching its lowest point at the peak of inflammation, and returning to normal levels as the inflammation resolved, indicating a role of PGD_2_ in the promotion of the resolution process. Similarly to PGE_2_, PGD_2_ can undergo spontaneous dehydrations, leading to the formation of 15-deoxy-Δ^12,14^-PGJ_2_ (15d-PGJ_2_), which can also act via DP. However this PG acts mainly via intracellular receptors, activating PPAR-γ and inhibiting nuclear factor kappa B (NF-κB). 15d-PGJ_2_ has anti-inflammatory and pro-resolution effects, inhibiting the secretion of IL-6, IL-1β, IL-12 and TNF-α from macrophages, and downregulating the production of inducible nitric oxide synthase (iNOS) [36]. 15d-PGJ_2_ is a very unstable molecule; its intermediate Δ^12^-PGJ_2_ is formed by the dehydration of PGD_2_ catalyzed by human serum albumin, which may bind and stabilize Δ^12^-PGJ_2_, as well as 15d-PGJ_2_ [36]. In our metabonomics analysis, no significant hits for 15d-PGJ_2_ were observed in BT and LL sera. However, the levels of compounds with an *m/z* potentially corresponding to Δ^12^-PGJ_2_ (*m/z* [M-H]^−^ 333.20712) were significantly higher in LL patients, and were reduced after MDT.

As mentioned above, the higher levels of LXA_4_, the predominant endogenously-generated LX, in leprosy patients suggested by the metabonomics analysis were confirmed by EIA. LXs are trihydroxytetraene-containing AA metabolites that are produced by at least 3 distinct LO pathways, involving interactions among diverse cell types, including leukocytes, epithelia, endothelia, and platelets. LXA_4_ and/or its aspirin-triggered isomer, 15-epi-LXA_4_ have a number of reported *in vitro* activities, including: (a) inhibition of neutrophil chemotaxis, adherence, transmigration, and activation; (b) suppression of the production of diverse chemokines by epithelial cells and leukocytes; (c) inhibition of IL-12 production by dendritic cells; (d) upregulation of monocyte chemotaxis and ingestion of apoptotic neutrophils; and (e) suppression of MMP production, while stimulating production of tissue inhibitors of MMPs. *In vivo*, LXA_4_ has been shown to have broad counter-regulatory properties, suppressing proinflammatory responses (preventing neutrophil-mediated damage, promoting the resolution of neutrophil-mediated inflammation), Th_2_-polarized responses (inhibiting inflammation and airway hyperresponsiveness in experimental asthma), and Th_1_ responses (suppressing immunopathology during infection with *Toxoplasma gondii*) alike [20], [37]. Moreover, LXA_4_ stimulates phagocytosis and IL-10 production in macrophages [38], a phenotype characteristic of foamy macrophages present in LL lesions [25].

Our metabonomics data on omega-3 PUFAs are sustained by a recent serum metabonomic analysis on leprosy patients, which showed a significant raise in the levels of EPA and DHA in sera from high-BI patients [39]. Also, hits that may correspond to DHA and DPA (a 22-carbon derivative of EPA) were detected in higher levels in skin lesions of LL patients when compared to BT lesions, reinforcing these data. Moreover, the remarkable differences in the levels of several potential omega-3 PUFA metabolites observed in leprosy patients before and after MDT, point to the participation of these bioactive lipid mediators in the immunopathology of leprosy. The anti-inflammatory properties of omega-3 PUFAs have been recently shown to be mediated, at least in part, by a new family of pro-resolving lipid mediators that include Rvs, PD1 and MaR (reviewed in [20]). Our metabonomics data showed the decrease of *m/z* that may correspond to RvE1 and RvE2, RvD1–4, RvD5–6, PD1, as well as MaR1 after treatment. Indeed, high levels of RvD1 were found by EIA in serum samples of leprosy patients, which returned to normal levels after treatment. Moreover, an *m/z* that corresponds to DPA was found in levels 50 times higher in skin biopsies of LL when compared to BT lesions.

Lipid mediators are produced in a temporally orchestrated fashion during inflammation. During the initial phases of inflammation, pro-inflammatory eicosanoids such as PGE_2_, PGD_2_ and LTB_4_ are generated. With time, a class-shift occurs towards anti-inflammatory and pro-resolving mediators (LXA_4_, 15d-PGJ_2_, Rvs, PD1 and MaR) that switch the inflammatory response off and restore homeostasis. Resolution of inflammation and return to homeostasis is actively mediated by these compounds and the failure of resolution is considered as one of the causes of chronic inflammatory diseases such as age-related macular degeneration, asthma, lupus erythematosus, atherosclerosis, chronic pulmonary disease, inflammatory bowel disease, multiple sclerosis, rheumatic arthritis and cancer [40]. In all of these cases, LX deficiency in association with high levels of pro-inflammatory mediators has been implicated in disease pathogenesis. Thus, LXA_4_ and its more stable synthetic analogues, as well as Rvs, PD1 and MaR and their agonists have emerged as novel therapeutic candidates via accelerated resolution of inflammation for the management of a broad range of disorders with an inflammatory component, including type 2 diabetes and cardiovascular diseases [41], [42]. On the other hand, production of LXA_4_ early during inflammation was shown to delay resolution and, in the case of infection, promote pathogen persistence in the host. This is the case for infections with *M. tuberculosis* and *M. marinum*, where an imbalance between LXA_4_ and pro-inflammatory eicosanoids (PGE_2_ and LTB_4_) during the early stages of infection has been shown to favor pathogen survival and multiplication [43].

Interestingly, a recent study on metabolic profiling of sera from tuberculosis (TB) patients also provided evidence for anti-inflammatory metabolic changes in this disease [44]. The authors found increased levels of kynurenine, the product of tryptophan catabolism by indoleamine 2,3 dioxygenase 1 (IDO1), in patients with active TB. This was significantly correlated with similarly increased abundance of the immunosuppressive stress hormone cortisol.

The metabonomics analysis presented herein discloses potential host tolerance mechanisms to *M. leprae* infection. Recently, the concept of disease tolerance as a defense strategy to infection has been introduced in the field of animal immunity (reviewed in [45]). While the immune system protects from infections primarily by detecting and eliminating the pathogen, tolerance does not directly affect pathogen burden, but rather, decreases immunopathology caused by the pathogens or the immune responses against them. Particularly the lepromatous pole of leprosy seems to be an excellent model to study disease tolerance in humans. Clinical data indicate that LL patients have developed tolerance mechanisms that allow them to survive with minimal pathology, despite the high bacterial burden. In LL patients, failure of the immune system to kill or inhibit *M. leprae* allows the mycobacteria to reproduce to very high numbers reaching multiple tissues and organs in a systemic infection. Heavy bacteremia is often observed in these patients but, in contrast to other bacterial infections, no symptoms of septicemia are observed. Moreover, a subtype of LL, known as diffuse LL, “pretty leprosy” or Lucio leprosy, appears in the earlier stages of disease as uniformly diffused, shiny infiltrations of all the skin of the body, without any actual lesions [46]. Increased tolerance to tissue damage can be achieved, in general, through tissue protection and repair. It is, therefore, reasonable to speculate that the higher levels of LXA_4_, and PGE_2_ levels, in association with the omega-3 PUFAs DHA, EPA, RvD1, and other potential Rvs, PD1 and MaR detected in leprosy patients may contribute to the molecular mechanisms that restrain the inflammatory responses in LL and at the same time favor *M. leprae* growth and persistence in the host. Indeed, the ameliorative effects of LXA_4_ and omega-3 PUFA metabolites have been reported in animal models of sepsis and through the observation of their inhibitory effects on the inflammatory response to endotoxin in humans (reviewed in [42], [47], [48]). Although the role of these resolving lipid mediators is well established in acute infections, more detailed studies on chronic infections are needed to establish the function of these mediators in determining disease outcome. Deciphering the molecular details of tolerance mechanisms in leprosy may pave the way to new prevention and management strategies of leprosy reactions as well as new treatments for many human maladies, including infectious, inflammatory and autoimmune diseases.

## Supporting Information

Figure S1 **The differential effect of leprosy clinical forms on arachidonic acid metabolism.** Schematic overview of the arachidonic acid metabolic pathway (adapted from http://www.genome.jp/kegg/). Metabolites in red are those that presented higher relative intensities in LL than in BT sera. Metabolites in black were not detected or were not affected over 2-fold. Detected *m/z* [M-H]^−^ values from affected metabolites are shown in parentheses. PG, prostaglandin; LT, leukotriene; TX, thromboxane; EET, epoxyeicosatrienoic acid; oxo-ETE, oxoicosatetraenoic acid; HETE, hydroxyeicosatetraenoic acid; HPETE, hydroperoxyeicosatetraenoic acid; DHET, dihydroxyeicosatrienoic acid; THETA, trihydroxyicosatrienoic acid.
(EPS)

Figure S2 **The differential effect of leprosy clinical forms on linoleic acid metabolism.** Schematic overview of the linoleic acid metabolic pathway (adapted from http://www.genome.jp/kegg/). Metabolites in red are those that presented higher relative intensities in LL than in BT sera. Metabolites in black were not detected or were not affected over 2-fold. Detected *m/z* [M-H]^−^ values from affected metabolites are shown in parentheses. EpOME, epoxyoctadecenoic acid; HPODE, hydroperoxyoctadecadienoic acid; HODE, hydroxyoctadecadienoic acid; TriHOME, trihydroxyoctadecenoic acid; DHOME, dihydroxyoctadecenoic acid; DiODE, dihydroxyoctadecadienoic acid; oxoODE, oxooctadecadienoic acid.
(EPS)

Figure S3 **The differential effect of leprosy clinical forms on omega-3 PUFA metabolism.** Schematic overview of omega-3 PUFA metabolism (adapted from http://www.genome.jp/kegg/). E-series resolvins, D-series resolvins, protectins, and maresin metabolic pathways adapted from [49] are shown. Metabolites in red are those that presented higher relative intensities in LL than in BT sera. Detected *m/z* [M-H]^−^ values from affected metabolites are shown in parentheses. Solid arrows, direct steps; dashed arrows, multiple steps that are not shown. EPA, eicosapentaenoic acid; DHA, docosahexaenoic acid; ETA, eicosatetraenoic acid; HEPE, hydroxyeicosatetraenoic acid; HpDHA, hydroperoxydocosahexaenoic acid; RvE, resolvin E; RvD, resolvin D; (N)PD1, (neuro)protectin D; MaR, maresin. *m/z* with relative changes that were close to 2-fold: *DHA = 1.99, **RvE1 = 1.93, ***ETA = 1.98. Fatty acids that can be obtained from the diet are indicated.
(EPS)

Figure S4 **The impact of MDT on arachidonic acid metabolism of LL and BT patients.** Schematic overview of arachidonic acid metabolism (adapted from http://www.genome.jp/kegg/). Metabolites in green are those that presented lower relative intensities after MDT in both BT and LL sera, and in red are those that presented lower relative intensities after MDT only in LL sera. No metabolites showed reduced abundances after MDT in BT sera only. Metabolites in black were not detected or were affected below the 2-fold cut-off. Detected *m/z* [M-H]^−^ values from affected metabolites are shown in parentheses. PG, prostaglandin; LT, leukotriene; TX, thromboxane; EET, epoxyeicosatrienoic acid; HETE, hydroxyeicosatetraenoic acid; HPETE, hydroperoxyeicosatetraenoic acid; DHET, dihydroxyeicosatrienoic acid.
(EPS)

Figure S5 **Circulating levels of eicosanoids on leprosy patients after MDT.** Box-plots represent the serum levels of PGD_2_ (a), PGE_2_ (b), LTB_4_ (c) and LXA_4_ (d) assessed in healthy controls, borderline tuberculoid patients (BT) after MDT and lepromatous leprosy patients (LL) after MDT. Median values are indicated by lines. Outliers were detected using the Grubbs' test and removed. Group comparisons were evaluated with Kruskall–Wallis non-parametric analysis of variance (ANOVA) and Dunn's multiple-range post hoc test. PGD_2_, prostaglandin D_2_; PGE_2_, prostaglandin E_2_; LTB_4_, leukotriene B_4_; LXA_4_, lipoxin A_4_. *P*-values higher than 0.05 are not shown.
(EPS)

Table S1 **Overview of DI-FT-ICR-MS results from leprosy patients sera.**
(XLSX)

Table S2 **Raw DI-FT-ICR-MS data of serum samples, negative ionization.**
(XLSX)

Table S3 **Raw DI-FT-ICR-MS data of serum samples, positive ionization.**
(XLSX)

Table S4 **Raw DI-FT-ICR-MS data of skin samples, negative ionization.**
(XLSX)

Table S5 **Raw DI-FT-ICR-MS data of skin samples, positive ionization.**
(XLSX)
